# Supplementary material for: Evaluation of sustainability of mycorrhizal industry development in Fujian Province-based on a combination of CRITIC empowerment method and cloud model
Source: PLoS One. 2023 Dec 21;18(12):e0281619. doi: 10.1371/journal.pone.0281619 (PMC10735044; doi:10.1371/journal.pone.0281619)
Supplement: Attachment — Submitted filename: Response to Reviewers.docx [file pone.0281619.s001.docx]

Response to Reviewers

In response to the editor's request, the authors responded as follows.

1. The format of the paper has been revised and adjusted.

2. After discussion with the co-authors, it was decided not to publish the paper in the Sustainable Development and Circular Economy collection, and this has been added in the cover letter.

3. The data used in the article and the corresponding calculation methods and sources are available in Table 1 of the article, and have been added at the bottom of Table 1 of the article.

4. Funding information has been updated.

5. The statement of data availability has been supplemented.

6. ORCID iD has been added.

7. The author information has been supplemented.

8.The author information has been supplemented.

The rest of the requirements have also been revised, if there is still that part is not done, I hope the editor can give the opportunity to revise, thank you very much!

Response to the suggestion of the first reviewer.

1. The strengths and weaknesses of the method and the scope of application have been added in the article. Supplemented in the conclusion future scope and limitations section.

**Future scope and limitations of the model**

As an evaluation method, the cloud model can be used alone or in combination with the assignment method, especially in DEA measurement related studies, which often calculate efficiency values after constructing a set of input-output indicators, and the measured efficiency values are often simply analyzed using charts and graphs, while the cloud model is able to fill the missing evaluation part in DEA related studies, and can conduct more in-depth data The cloud model is able to fill the missing evaluation part of DEA related research, and can analyze, compare and explore the data in more depth. It can enrich the research content of DEA measurement articles, so that the research content can be expanded into two major parts: measurement and evaluation. Cloud model as an evaluation method is also applicable to evaluate various scientific problems. Its application scope is relatively wide. However, the cloud model itself belongs to the research methods of engineering disciplines, and the use of cloud model in the social science field is still relatively scarce, and the application of cloud model in the social science field still has a lot of room for exploration. And the cloud model is more suitable for the comparison between two indicators, that is, through the evaluation of the cloud model to solve the problem of which indicator results are better. Its main function is to realize the mutual transformation of qualitative and quantitative, and it is more suitable for the evaluation and comparison of multi-dimensional and multi-indicators, while the evaluation of single indicator and single system may be contrary to the original purpose of the cloud model.

2. The literature review has been supplemented with emphasis on the application of the empowerment method and cloud model. And the articles recommended by the reviewers are cited.

The current academic research on multi-criteria decision making often uses the assignment method to divide the weights of multiple indicator systems in order to achieve the scientific nature of the evaluation results. The mainstream view is that the assignment method can be divided into two types: subjective assignment method and objective assignment method, and subjective assignment method, such as AHP method [23], FUCOM method [24], Delphi method, etc., which mainly relies on the information provided by experts to assign weights to indicators, and its shortcomings are obvious. This means that the decision maker influences the decision making process and the outcome is more influenced by the decision maker's subjective preferences. The objective empowerment method uses existing data to make the empowerment. In comparison, the advantage of the objective assignment method over the subjective assignment method is that it is not influenced by the decision maker's preferences and is more scientific and reliable in the representation of the evaluation results [25]. Although scholars have pointed out that the objective assignment methods such as entropy method and CRITIC assignment method have the same defects in practice, CRITIC assignment method is more systematic than entropy method because it not only considers the comparison intensity between indicators but also considers the conflict between indicators, and has the advantages of both entropy method and principal component analysis.The use of CRITIC assignment method can The use of CRITIC weighting method can reflect the evaluation results more accurately and objectively and scientifically [26]. The advantage of the cloud model is that it can simulate the overall results through multiple simulations, reflect the stability and scientific nature of the overall results, and can realize the mutual transformation of quantitative and qualitative through its own data. Compared with the common line graphs and other ways to interpret the evaluation results, the evaluation of the cloud model can be more scientific and reliable, and it can objectively analyze the good and bad evaluation results and visualize the results, so that it is easy to analyze and dig into the overall situation of the development of the evaluation results, the overall stability, the differences in development, the stability of the development differences, and many other information. In the current research, the empowerment method is often combined with the cloud model, and the common combination is mainly the entropy method and cloud model, and the combination of CRITIC empowerment method and cloud model. Based on the introduction of the empowerment method above and considering the main combination of scholars at present, the study adopts the combination model of CRITIC empowerment method and cloud model to analyze and evaluate the sustainability of mycorrhizal industry.

3. The article is less researched in mycorrhiza and mycorrhiza industry, so there are not many literature that can be referenced and cited. Therefore, some of the cited literature are published in earlier years, but they still provide great reference value for the research of mycorrhizal industry, so the authors did not delete them, but the authors listened to the advice of the reviewer and cited the relevant literature provided by the reviewer to supplement the content of the article.

Response to the second reviewer's suggestion.

The article has taken the expert's advice and the introduction has been revised as follows.

1. The introduction has been modified and supplemented.

2. The introduction supplements the innovative explanation of the article.

3.The literature review is supplemented, focusing on the application of the empowerment method and cloud model and the analysis and interpretation of the empowerment method by related studies. The articles recommended by the reviewers are also cited.

**Future scope and limitations of the model**

As an evaluation method, the cloud model can be used alone or in combination with the assignment method, especially in DEA measurement related studies, which often calculate efficiency values after constructing a set of input-output indicators, and the measured efficiency values are often simply analyzed using charts and graphs, while the cloud model is able to fill the missing evaluation part in DEA related studies, and can conduct more in-depth data The cloud model is able to fill the missing evaluation part of DEA related research, and can analyze, compare and explore the data in more depth. It can enrich the research content of DEA measurement articles, so that the research content can be expanded into two major parts: measurement and evaluation. Cloud model as an evaluation method is also applicable to evaluate various scientific problems. Its application scope is relatively wide. However, the cloud model itself belongs to the research methods of engineering disciplines, and the use of cloud model in the social science field is still relatively scarce, and the application of cloud model in the social science field still has a lot of room for exploration. And the cloud model is more suitable for the comparison between two indicators, that is, through the evaluation of the cloud model to solve the problem of which indicator results are better. Its main function is to realize the mutual transformation of qualitative and quantitative, and it is more suitable for the evaluation and comparison of multi-dimensional and multi-indicators, while the evaluation of single indicator and single system may be contrary to the original purpose of the cloud model.

4.The subjective weighting method often leads to the subjectivity of the results. The objective weighting method is based on the data itself to obtain the corresponding weights, which can make the overall results more objective and reliable.

5.At present, the research mostly combines CRITIC weighting method with cloud model. This research also adopts the approach of most articles and chooses to use CRITIC weighting method with cloud model.

6.The article is less researched in mycorrhiza and mycorrhiza industry, so there are not many literature that can be referenced and cited. Therefore, some of the cited literature are published in earlier years, but they still provide great reference value for the research of mycorrhizal industry, so the authors did not delete them, but the authors listened to the advice of the reviewer and cited the relevant literature provided by the reviewer to supplement the content of the article.

7.The conclusion section is supplemented with the future scope and limitation section, focusing on the advantages and disadvantages of the modeling approach.

The current academic research on multi-criteria decision making often uses the assignment method to divide the weights of multiple indicator systems in order to achieve the scientific nature of the evaluation results. The mainstream view is that the assignment method can be divided into two types: subjective assignment method and objective assignment method, and subjective assignment method, such as AHP method [23], FUCOM method [24], Delphi method, etc., which mainly relies on the information provided by experts to assign weights to indicators, and its shortcomings are obvious. This means that the decision maker influences the decision making process and the outcome is more influenced by the decision maker's subjective preferences. The objective empowerment method uses existing data to make the empowerment. In comparison, the advantage of the objective assignment method over the subjective assignment method is that it is not influenced by the decision maker's preferences and is more scientific and reliable in the representation of the evaluation results [25]. Although scholars have pointed out that the objective assignment methods such as entropy method and CRITIC assignment method have the same defects in practice, CRITIC assignment method is more systematic than entropy method because it not only considers the comparison intensity between indicators but also considers the conflict between indicators, and has the advantages of both entropy method and principal component analysis.The use of CRITIC assignment method can The use of CRITIC weighting method can reflect the evaluation results more accurately and objectively and scientifically [26]. The advantage of the cloud model is that it can simulate the overall results through multiple simulations, reflect the stability and scientific nature of the overall results, and can realize the mutual transformation of quantitative and qualitative through its own data. Compared with the common line graphs and other ways to interpret the evaluation results, the evaluation of the cloud model can be more scientific and reliable, and it can objectively analyze the good and bad evaluation results and visualize the results, so that it is easy to analyze and dig into the overall situation of the development of the evaluation results, the overall stability, the differences in development, the stability of the development differences, and many other information. In the current research, the empowerment method is often combined with the cloud model, and the common combination is mainly the entropy method and cloud model, and the combination of CRITIC empowerment method and cloud model. Based on the introduction of the empowerment method above and considering the main combination of scholars at present, the study adopts the combination model of CRITIC empowerment method and cloud model to analyze and evaluate the sustainability of mycorrhizal industry.

8. In response to the expert's suggestion to add in-depth calculations in the case study section, I am very sorry that the authors did not understand the expert's suggestion well. If necessary, we hope that the experts can provide more detailed comments, and the authors will definitely learn from them and make changes. Also, the authors would like to clarify that the overall analysis is based on the paradigm of the study, and that the overall content that needs to be presented and reported has been reported and presented.

9. The problem of adopting this method is of great social and management significance. Need to include how this approach addresses these impacts.

It has been supplemented and explained in the discussion section.

10.The literature review is supplemented, focusing on the application of the empowerment method and cloud model and the analysis and interpretation of the empowerment method by related studies. The articles recommended by the reviewers are also cited.

Future scope and limitations of the model

As an evaluation method, the cloud model can be used alone or in combination with the assignment method, especially in DEA measurement related studies, which often calculate efficiency values after constructing a set of input-output indicators, and the measured efficiency values are often simply analyzed using charts and graphs, while the cloud model is able to fill the missing evaluation part in DEA related studies, and can conduct more in-depth data The cloud model is able to fill the missing evaluation part of DEA related research, and can analyze, compare and explore the data in more depth. It can enrich the research content of DEA measurement articles, so that the research content can be expanded into two major parts: measurement and evaluation. Cloud model as an evaluation method is also applicable to evaluate various scientific problems. Its application scope is relatively wide. However, the cloud model itself belongs to the research methods of engineering disciplines, and the use of cloud model in the social science field is still relatively scarce, and the application of cloud model in the social science field still has a lot of room for exploration. And the cloud model is more suitable for the comparison between two indicators, that is, through the evaluation of the cloud model to solve the problem of which indicator results are better. Its main function is to realize the mutual transformation of qualitative and quantitative, and it is more suitable for the evaluation and comparison of multi-dimensional and multi-indicators, while the evaluation of single indicator and single system may be contrary to the original purpose of the cloud model.
